# Egg freezing in transgender men

**DOI:** 10.5935/1518-0557.20260009

**Published:** 2026

**Authors:** Caiane Gabrielle Souza de Paula, Paula Bruno Monteiro, Raissa de Vasconcelos Cavalcanti

**Affiliations:** 1 Unichristus Centro Universitário Christus. Fortaleza, CE, Brazil

**Keywords:** fertility preservation, transmasculine male, hormone therapy, oocyte quality, cryopreservation

## Abstract

Transgender men are people who were biologically assigned female at birth but identify as men. It is important to consider the impacts of testosterone and reassignment surgery on fertility. Hormone therapy can compromise ovarian function, and surgery can result in the irreversible loss of reproductive capacity. Fertility preservation has emerged as a viable option for those who wish to have biological children. This study aimed to evaluate the effectiveness of this method in trans men, analyze the ovarian response to hormonal stimulation, and investigate the impact of the duration of therapy on the quality and quantity of oocytes. PubMed, SciELO, and LILACS databases were consulted, with articles from 2015 to 2025 in English, Portuguese, and Spanish, following the PRISMA guide. The data was organized in Microsoft Excel tables. Case, cohort, cross-sectional, prospective, and retrospective studies were included, excluding articles out of scope, unavailable, used animals, and reviews. Initially, 404 articles were identified, with four duplicates excluded. After screening, 25 articles remained for full reading, of which 13 were analyzed. The duration of hormone therapy varied between 2 and 12 years among the patients in the studies analyzed, and only one patient did not have sufficient data due to the absence of embryo transfer. The studies showed promising results for egg cryopreservation, even with prolonged use of testosterone, but there are still no definitive conclusions about the long-term effect on fertility. This review contributes to the understanding of fertility in trans men and their reproductive alternatives.

## INTRODUCTION

The transgender male population in Brazil has gained visibility through social movements, driven by the creation of associations and institutes (Pereira *et al.*, 2021). A transgender man is someone who identifies as male, but was biologically assigned female at birth, regardless of whether he is undergoing procedures or interventions to modify his body (Okano *et al.*, 2022). However, transsexual men who undergo sex reassignment surgery lose their ovarian function and become unable to undergo pregnancy. Furthermore, despite losing their reproductive capacity after surgery, many retain the desire for parenthood (Wierckx *et al.*, 2012).

On the other hand, transgender men who take testosterone hormone therapy but have not undergone gender affirmation surgery retain their reproductive capacity. However, the effects of testosterone on the ovaries are not yet conclusive, making fertility preservation counseling essential before hormonal or surgical treatment (Gale *et al.*, 2021). Theoretically, the use of exogenous hormones in gender affirming hormone therapy suppresses the effect of the pituitary gland, the gland responsible for releasing follicle stimulating hormone (FSH) and luteinizing hormone (LH), on which oocyte maturation depends. However, LH and FSH are not completely inhibited and oocyte maturation is not always impaired during hormone replacement treatment with testosterone (Stolk *et al.*, 2023). Testosterone is available in patches, injections and gels, and the effects include increased muscle mass, hair growth, clitoral enlargement and amenorrhea. It is therefore important to mention that transgender men can start hormone treatment with testosterone from the age of 16 and undergo sex reassignment surgery at the age of 21, as long as they present a psychological or psychiatric report, have been on hormone treatment for at least two years and understand the risks and benefits of surgery (Lara *et al.*, 2023).

It is important to note that gender affirmation therapy is seen by transgender men as a real relief from mental suffering, since they feel inadequate with the body they have due to the gender they identify with (Pereira *et al.*, 2021). Transgender men who have not opted for sex reassignment surgery, while retaining their reproductive organs, can preserve their fertility through oocyte cryopreservation, since this technique allows germ cells to be frozen at low temperatures, preserving them for future use (Mattawanon *et al.*, 2018).

However, the technique of oocyte cryopreservation was first reported in the 1980s, but it was only with the advent of vitrification in the mid-2000s that improvements in this technique were noticed (Iussig *et al.*, 2019). Currently, vitrification is the preferred technique for oocyte cryopreservation, as this approach involves solidification at temperatures below the glass transition temperature, increasing viscosity. Oocyte survival rates as well as implantation and developing pregnancy rates are high, which gives vitrification high credibility (Cavagna *et al.*, 2020). This technique allows for the cryopreservation of oocytes without the formation of ice crystals, unlike the technique pioneered in the 80s. The literature attests that oocytes that have been vitrified and then fertilized survive the procedure with good embryonic development (Chang *et al.*, 2022).

Transgender men who choose to preserve fertility through oocyte cryopreservation should be aware of some possible challenges and have perseverance throughout the process, since the procedure of collecting and vitrifying eggs requires hormonal treatment for ovarian stimulation and an invasive technique for collecting these oocytes (Mitu, 2016). After the oocyte freezing procedure, assisted reproduction techniques can be carried out with transgender men who have not yet undergone the gender affirmation surgical process or opt for a surrogate uterus (Cheng *et al.*, 2019).

In addition, freezing oocytes in transgender men who are on testosterone therapy is possible, but it comes with obstacles. It is necessary to stop taking testosterone for a period of time before ovarian stimulation, a difficult scenario for transgender men due to the gender dysphoria that can occur (Gale *et al.*, 2021). However, more studies are being carried out to assess the real need to stop hormone therapy for cryopreservation (Cho *et al.*, 2020).

With this in mind, this study aims to assess the feasibility of egg freezing in transgender men, analyze the ovarian response in transgender men who are on hormone therapy, compare the duration of hormone treatment and the impact on ovarian response, oocyte quantity and quality.

## METHODS

### Type of study

This study is a systematic review of the literature. Therefore, the *Preferred Reporting Items for Systematic Reviews and Meta-Analyses* (PRISMA) guidelines were used to carry it out.

### Search strategy

Articles from 2015 to 2025 were used with the descriptors selected by DeCS (Descriptors in Health Science). After selection, these descriptors were combined with Boolean operators in various searches: *fertility preservation* AND *transgender persons, hormone replacement therapy* AND *testosterone congeners, transgender persons* AND *testosterone* congeners, *ovarian reserve* AND *quality, hormone replacement therapy* AND *transgender persons, cryopreservation* AND *ovum*, in English, Portuguese and Spanish. The search strategy was carried out in three different databases: Pubmed, SciELO and Lilacs.

**Criteria for selecting studies**

**Inclusion *criteria***

- Cohort studies
- Cross-sectional studies
- Clinical studies
- Full articles
- Case reports
- Prospective studies
- Retrospective studies
- Qualitative studies

### Exclusion *criteria*

- Out-of-scope studies
- Review articles
- Articles with animals
- Review articles
- Duplicate articles
- Incomplete articles
- Paid items unavailable

### Data collection

The information collected from the articles was analyzed and organized using Microsoft Excel software to create tables with the data obtained.

### Data extraction

After collection, these articles were read to obtain the following data:

- Author’s name
- Year of publication
- Time on hormone therapy
- Ovarian respons;
- Number of eggs retrieved and vitrified after ovarian stimulation
- The desire to have children
- Ovarian quality
- Fertilization rate
- Blastocyst formation
- Pregnancy rate

## RESULTS

**Figure 1:**
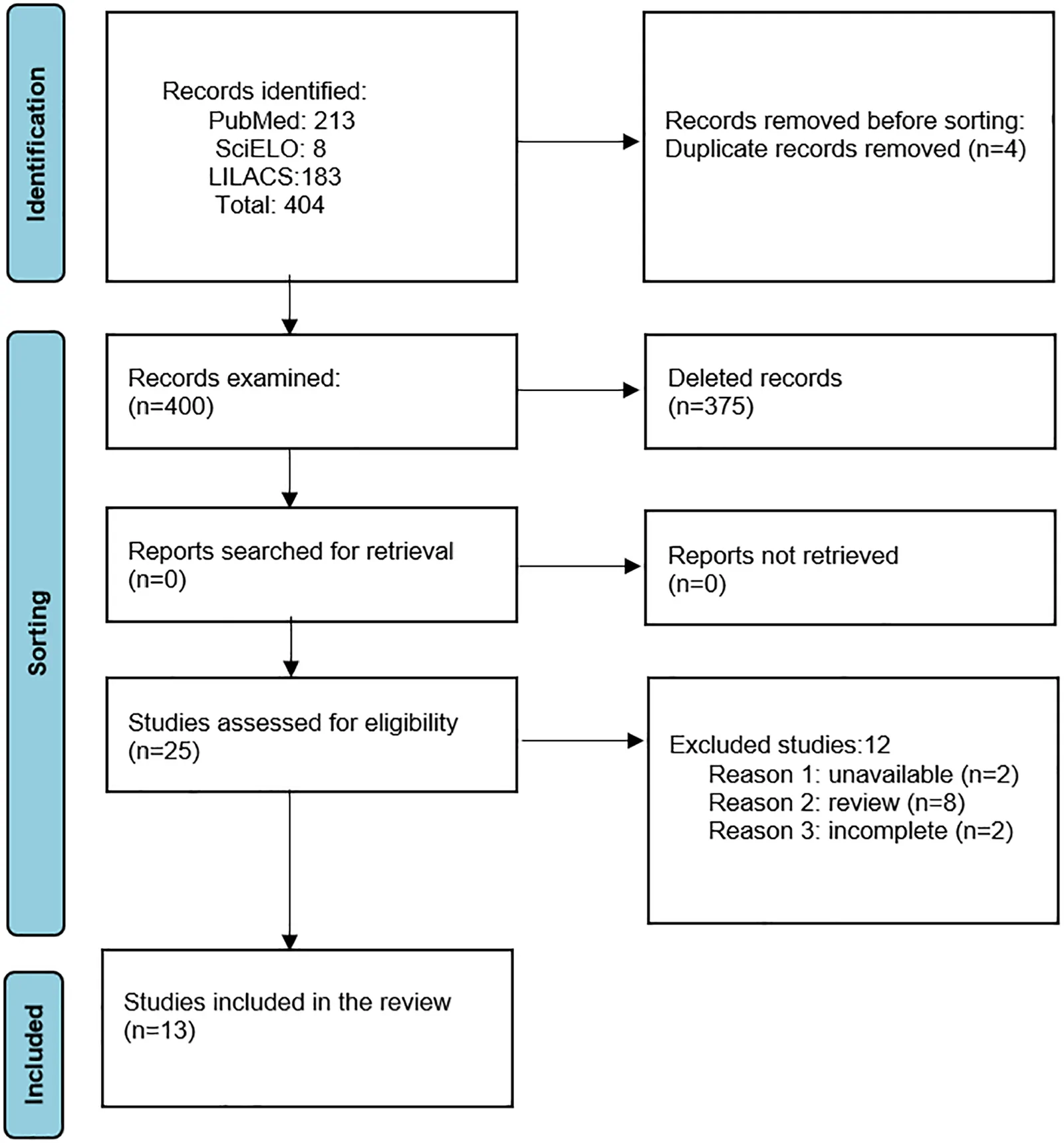
Flowchart of selected articles. Source: Preferred Reporting Items for Systematic Reviews and Meta-Analyses (PRISMA).

The records obtained through the data search were: PubMed (n=213), SciELO (n=8), LILACS (n=183), resulting in 404 articles in total. After the initial search, 4 duplicate articles were removed, leaving 400 articles for screening. After reading the title and abstract, and checking availability, 375 articles that did not fit the inclusion criteria were excluded, leaving 25 articles to be read in full. After full reading, 13 articles were analyzed and 12 were excluded because they were not available, were reviews or were incomplete (Figure 1).

A total of 148 transgender male patients were analyzed, considering both case reports and studies with larger samples. In some studies, however, the total number of the population evaluated was not specified. The average age of the participants ranged from 19 to 35 years. Three of the thirteen articles analyzed were case reports, in which 4 clinical cases were observed. Four transgender men were using testosterone hormone therapy, two patients had live births and no gestational complications, one patient had an ongoing pregnancy and another had no reported pregnancy, as there had been no embryo transfer by the time the article was published. However, this patient had 23 mature oocytes cryopreserved, of which 14 were successfully fertilized via ICSI with donor sperm. Eight embryos developed to the blastocyst stage; however, no transfer was carried out (Resende *et al.*, 2020; Hassan *et al.*, 2022; Moravek *et al.*, 2023). The studies found a preserved ovarian reserve, in addition to considerable amounts of recovered and mature oocytes, as well as a surprising vitality of the ovarian cells exposed to long-term testosterone, suggesting that the ovarian reserve may be resilient to testosterone in the long term (Amir *et al.*, 2020a; Marschalek *et al.*, 2020).

In addition, two studies, both prospective and one of them prospective and cross-sectional, showed a preserved ovarian reserve even with testosterone use, antimullerian within the normal range, preserved antral follicle count and 4 pregnancies. Of the 119 patients in these two studies, 4 pregnancies were observed with live births after the use of hormone therapy ranging from 3 to 12 years of testosterone. Despite the extended use of the hormone, it was possible to become pregnant and have biological children, suggesting that ovarian function remained sufficiently preserved to enable pregnancy (Yaish *et al.*, 2021; Minotti *et al.*, 2022). No article provided data on oocyte survival after thawing, but they suggest good post-thaw viability, especially when cryopreservation is carried out before or even during hormone therapy.

### Pregnancy and conception in transgender men on testosterone hormone therapy

Three of the thirteen articles selected for analysis are case reports, in which four transmasculine patients were on hormone therapy with testosterone. Two of these patients had live births with no gestational complications, one of whom became pregnant through sexual intercourse, i.e. without transferring the embryo to his cisgender female partner. A third patient had an ongoing pregnancy after the transfer of a blastocyst to his cis partner, while the fourth patient had no report of an ongoing pregnancy, as there had been no embryo transfer to date. Patient number 1, aged 26, had been using testosterone for 3 years and 8 months prior to controlled ovarian stimulation (COS). The stimulation lasted 16 days, resulting in the recovery of 14 oocytes, 13 of which were mature. Nine were fertilized with donor sperm using intracytoplasmic sperm injection (ICSI). Two blastocysts developed on days 5 (D5) and 6 (D6), and one euploid blastocyst was transferred, resulting in a live birth with no complications. Patient number 2, 34, had been on hormone therapy (HT) for 10 years and had not stopped taking androgens during SOC. This patient had 23 mature oocytes cryopreserved, of which 14 were successfully fertilized via ICSI with donor sperm. Eight embryos developed to the blastocyst stage, but no transfer was carried out (Moravek *et al.*, 2023). The third patient, also 34 years old, had been on TH with testosterone for 2 years and was advised to stop using the androgen. He had 16 oocytes retrieved, 12 of which were mature. A blastocyst was transferred to his cisgender female partner, and an ongoing pregnancy was detected (Resende *et al.*, 2020). Patient number 4, aged 21, had been on HT for over two years, but stopped using testosterone two months before becoming pregnant. Pregnancy was achieved through sexual intercourse with her cisgender male partner, and the transmasculine patient herself became pregnant, resulting in a live birth with no gestational complications (Hassan *et al.*, 2022) (Table 1).

**Table 1:** Case report summary of fertility preservation and reproductive outcomes in transmasculine individuals.

Author's name and year of publication	Study population	Time on hormone therapy	Ovarian response	Recovered and vitrified eggs	Ovarian quality	Fertilization rate	Blastocyst formation	Pregnancy rate
**Moravek et al. (2023)**	26-year-old transmasculine individual	3 years and 8 months	AMH: 8 ng/ml, AFC: 23, Stimulation: 16 days	14 eggs retrieved, 13 mature	-	9/13 (69%)	2 blastocysts biopsied and cryopreserved	1 blastocyst transferred, live birth
**Moravek et al. (2023)**	34-year-old transmasculine individual	10 years	AMH: 9 ng/ml, AFC: No data, Stimulation: No data	23 cryopreserved eggs, 14 fertilized	-	14/23 (61%)	8 cryopreserved blastocysts	No transfers made so far
**Resende et al. (2020)**	34-year-old transmasculine individual	2 years	No data, Stimulation: 12 days	16 eggs retrieved, 12 mature	-	12/12 (100%)	5 cryopreserved blastocysts, 1 transferred	Confirmed pregnancy
**Hassan et al. (2022)**	21-year-old transmasculine individual	2 years	No data, Stimulation: No data	No stimulation performed	-	-	-	Spontaneous pregnancy, cesarean birth
**Minotti et al. (2022)**	16 trans men, average age 22	6-12 months	AMH: 2.89 ng/ml	-	-	-	-	-
**Morong et al. (2022)**	Adult transgender population	-	-	-	-	-	-	-
**Yaish et al. (2021)**	Adult transgender men	Up to 12 years old	-	-	-	-	-	4 transgender men have fathered 7 biological children
**Marschalek et al. (2020)**	20 transgender men	In hormone therapy	No data	No data	Vital cells	-	-	-
**Amir et al., (2020a)**	9 trans men compared to 39 cis women	Before starting hormone therapy	Average AFC: 19.2	There was no significant difference between the two groups	-	-	-	-
**Amir et al., (2020b)**	12 transgender men, 6 without exposure to testosterone and 6 with exposure	Between 1 and 12 years old	Average AFC: 16.5	Ova retrieved average: 23 Only one patient vitrified oocytes: 11MII	-	Average: 57.5%	Average: 47.78%	Only one patient had ongoing successful pregnancy data
**Leung et al. (2019)**	26 Transmasculine individuals	-	-	19,9 ± 8,7	-	-	-	7 couples had transfers, obtaining live births
**De Roo et al. (2017)**	40 Transmasculine individual	More than a year	-	34.30% of oocytes in metaphase II were obtained	-	-	-	-
**Chen et al. (2017)**	13 transgender teenagers (7 trans women and 6 trans men)	-	-	Only one patient cryopreserved oocytes	-	-	-	-
**Armuand et al. (2017)**	15 Adult transgender men referred for fertility preservation	-	-	On average 11 oocytes per individua	-	-	-	-

### Analysis of the ovarian response in transgender men using testosterone

Two articles, both prospective and cross-sectional studies, attest to the fact that ovarian reserve can be preserved even with the use of testosterone in gender affirmation therapy. The prospective study was carried out with 16 transgender men and assessed the level of anti-müllerian hormone (AMH) during short-term androgen treatment. AMH levels did not differ significantly before and after 6 to 12 months of hormone therapy (Minotti *et al.*, 2022). In the prospective, cross-sectional study, it was observed that AMH decreases rapidly with HT, but remains within the normal range, which is suggestive of preserved ovarian reserve. In the prospective portion of the study, 56 individuals took part, 27 of whom had polycystic ovary syndrome. There was a significant decrease in the whole group, but a new analysis was carried out separately on individuals with and without polycystic ovary syndrome (PCOS) and it was observed that the decline in AMH levels was explained by the age of the participants with PCOS, while in patients without PCOS, AMH did not change at all during the study. In the cross-sectional part of the study, 47 individuals were selected, all of whom were on constant testosterone treatment, the average duration of treatment being 35 months. AMH levels correlated inversely with age, but not with treatment duration. The antral follicle count (AFC) was preserved, as it was not associated with age or duration of treatment. Four men experienced pregnancies and had biological children after being treated with testosterone for a period of between 3 and 12 years (Yaish *et al.*, 2021) (Table 1).

### Feasibility of egg freezing in transgender men

One retrospective and one cross-sectional study attested that the main barriers to fertility preservation for oocyte cryopreservation are the cost of the procedures and the interruption of hormone therapy, since the androgen break causes gender dysphoria and delays the transition (Chen *et al.*, 2017; Morong *et al.*, 2022). In addition, a qualitative study of 15 transgender men aged between 19 and 35 who were going to undergo fertility preservation observed that stopping testosterone, together with the genital examinations that need to be carried out, culminated in gender dysphoria and incongruence (Armuand *et al.*, 2017). On the other hand, another 3 articles attest to the efficacy of egg cryopreservation for fertility preservation, this being a viable procedure before hormonal treatment with testosterone and during, as they show an excellent response to ovarian stimulation (Leung *et al.*, 2019; Amir *et al.*, 2020a; 2020b). In addition, 2 other articles prove the surprising vitality of ovarian cells even after prolonged exposure to testosterone (De Roo *et al.*, 2017; Marschalek *et al.*, 2020) (Table 1).

### The duration of hormone treatment and its impact on ovarian response, oocyte quantity and quality

Of the 13 articles analyzed, the duration of hormone treatment for trans men ranged from 6 months to 12 years. Only 1 patient did not have sufficient data, as no transfer had taken place by the time the article was published. However, this patient had blastocysts resulting from cryopreserved oocytes (Moravek *et al.*, 2023). The studies analyzed positively addressed the relationship between hormone therapy time and ovarian response, attesting to a preserved ovarian reserve, in addition to considerable quantities of recovered and mature oocytes, as well as a surprising vitality of the ovarian cells exposed, in the long term, to testosterone (De Roo *et al.*, 2017; Amir *et al.*, 2020a; 2020b; Marschalek *et al.*, 2020; Yaish *et al.*, 2021; Minotti *et al.*, 2022) (Table 1).

## DISCUSSION

Thirteen articles analyzed provided relevant evidence regarding reproductive capacity, ovarian response, the impact of hormone treatment and the barriers faced by transgender men in accessing fertility preservation. This review identified that the studies analyzed point to a positive association between testosterone use and ovarian response, with a preserved ovarian reserve, ovarian cell vitality even with androgen use and an unchanged antral follicle count (Yaish *et al.*, 2021; Minotti *et al.*, 2022). Furthermore, these analyses were confirmed, as no effects of hormone therapy were found on antimüllerian levels and antral follicle counts in trans men (De Roo *et al.*, 2017; Borrás *et al.*, 2022).

Transgender men’s desire for biological parenthood is increasingly present, although there are obstacles to be faced (Gale *et al.*, 2021; Ghofranian *et al.*, 2024). Another interesting fact is that other studies have shown that stopping hormone treatment with testosterone is seen as a real villain by these individuals, due to the intensification of gender dysphoria (Chen *et al.*, 2017; Morong *et al.*, 2022). However, the literature states that prolonged testosterone interruption is not necessarily a requirement for successful fertility preservation. An example of this is the case of a 28-year-old transgender man who, after three years on testosterone, stopped treatment for just 24 days and had 13 eggs retrieved, 11 of which were mature and vitrified (Cho *et al.*, 2020).

The total absence of testosterone interruption is also feasible. This review identified one case report in which it was possible for a trans man to obtain 23 cryopreserved mature oocytes without interrupting the 10-year hormone therapy during controlled ovarian stimulation. However, two other case reports resulted in live births, one of which occurred by interrupting hormone treatment of more than 2 years for 2 months and the other by interrupting hormone therapy of almost 4 years (Resende *et al.*, 2020; Hassan *et al.*, 2022; Moravek *et al.*, 2023).

In contrast, Adeleye *et al.* (2019) observed that between two groups of trans men - with and without a history of testosterone - the number of oocytes recovered was higher in the group without previous hormone therapy (average of 25.5 oocytes) compared to the group with a history of use (average of 12 oocytes). Despite this, three pregnancies with oocytes from trans men using testosterone were successful. On the other hand, Amir *et al.* (2020b) made a comparison with a group of 12 transgender men, six of whom had not been exposed to testosterone and another six of whom had used testosterone as a hormone therapy. This comparison showed that there was no significant difference in the number of oocytes retrieved, the number of mature oocytes and the oocyte maturity rate. In addition, five of the six trans men who used testosterone cryopreserved their embryos and obtained good quality embryos. Most of the studies showed that ovarian function was preserved and that significant quantities of mature oocytes were obtained, even after periods of 2 to 12 years of hormone therapy. This reinforces the hypothesis that prolonged exposure to testosterone does not necessarily compromise fertility. However, the lack of conclusive data on the long-term effects of testosterone prevents definitive statements.

The feasibility of cryopreserving oocytes in transgender men has been confirmed in several studies that have reported a good response to ovarian stimulation even after prolonged exposure to testosterone (Leung *et al.*, 2019; Amir *et al.*, 2020a; 2020b), and this technique is considered a significant advance in *in vitro* fertilization (Casciani *et al.*, 2023). Even so, important barriers have been identified, such as the high costs of the procedures and, above all, the emotional impact of stopping hormone therapy, which can aggravate gender dysphoria. The need for invasive gynecological examinations has also been pointed out as a factor triggering discomfort and gender incongruence (Armuand *et al.*, 2017; Chen *et al.*, 2017; Morong *et al.*, 2022).

It is important to highlight that the present study has some limitations. One of them is the limited number of available studies, which compromises the depth of the analyses and conclusions. It was also observed that many of the studies included in the analysis have small sample sizes, which limits the generalizability of the results. Another relevant point is the lack of a single or standardized methodology among the analyzed studies. Furthermore, there was significant variation in the duration of hormone therapy (HT) interruption, as well as considerable diversity in the characteristics of the studied populations. These limitations underscore the need for further research on the topic, with consistent methodologies and more robust samples.

## CONCLUSION

This study concludes that the use of testosterone does not prevent the preservation of fertility in transgender men through egg cryopreservation. It was also observed that hormone therapy with testosterone does not significantly compromise ovarian reserve or oocyte quality and quantity. Despite the scarcity of studies evaluating the long-term effects of androgens on the ovaries, cryopreservation appears to be a viable alternative, especially considering that many trans men choose to freeze their eggs and carry out embryo transfer. The case reports analyzed show the real possibility of conception even after prolonged use of testosterone, with reproductive success rates and live births from cryopreserved oocytes exposed to hormone therapy. A major limitation of this review was the small number of studies with large, representative samples, which limits the generalizability of the findings. It reinforces the need for further research with large samples in order to deepen knowledge on the subject and ensure that transgender men have equal, informed, and respectful access to reproductive technologies.
